# Karyotype and Phylogenetic Relationship Analysis of Five Varieties and Cultivars of *Zanthoxylum armatum* Based on Oligo-FISH

**DOI:** 10.3390/genes14071459

**Published:** 2023-07-17

**Authors:** Zhoujian He, Yuting Lei, Wei Gong, Meng Ye, Xiaomei Luo

**Affiliations:** 1 College of Forestry, Sichuan Agricultural University, Chengdu 611130, China; hezhouj@163.com (Z.H.); mihualyt@126.com (Y.L.); gongwei@sicau.edu.cn (W.G.); 2 National Forestry and Grassland Administration Key Laboratory of Forest Resources Conservation and Ecological Safety on the Upper Reaches of the Yangtze River & Forestry Ecological Engineering in the Upper Reaches of the Yangtze River Key Laboratory of Sichuan Province, Sichuan Agricultural University, Chengdu 611130, China

**Keywords:** *Zanthoxylum armatum*, karyotype, oligo-FISH, genetic relationship

## Abstract

Green prickly ash (*Zanthoxylum armatum*) has edible and medicinal value and is an economically significant plant in many countries. *Z. armatum* has many cultivars and varieties with similar phenotypes that are difficult to distinguish via traditional methods. In this study, we utilized oligo-FISH to distinguish five varieties and cultivars of *Z. armatum* on the basis of three oligonucleotide probes of 5S rDNA, (AG_3_T_3_)_3_, and (GAA)_6_. Karyotype analysis of the five varieties and cultivars of *Z. armatum* showed that the *Z. armatum* ‘Tengjiao’ karyotype formula was 2*n* = 2*x* = 98*m* with karyotype type 1C and an arm ratio of 4.3237, including two pairs of 5S rDNA signals and five pairs of (GAA)_6_ signals. The karyotype formula of *Z. armatum* ‘Youkangtengjiao’ was 2*n* = 2*x* = 128*m* + 8*sm* with karyotype type 2B and an arm ratio of 3.5336, including three pairs of 5S rDNA signals and 17 pairs of (GAA)_6_ signals. The karyotype formula of *Z. armatum* var. *novemfolius* was 2*n* = 2*x* = 134*m* + 2*sm* with karyotype type 1C and an arm ratio of 5.5224, including two pairs of 5S rDNA signals and eight pairs of (GAA)_6_ signals. The karyotype formula of *Z. armatum* ‘YT-03’ was 2*n* = 2*x* = 2*M* + 128*m* + 4*sm* + 2*st* with karyotype type 2C and an arm ratio of 4.1829, including three pairs of 5S rDNA signals and nine pairs of (GAA)_6_ signals. The karyotype formula of *Z. armatum* ‘YT-06’ was 2*n* = 2*x* = 126*m* + 10*sm* with cytotype 2B and an arm ratio of 3.3011, including three pairs of 5S rDNA signals and two pairs of (GAA)_6_ signals. The five varieties and cultivars of *Z. armatum* had (AG_3_T_3_)_3_ signals on all chromosomes. The chromosomal symmetry of *Z. armatum* ‘Tengjiao’ was high, whereas the chromosomal symmetry of *Z. armatum* 'YT-03' was low, with the karyotypes of the five materials showing a trend toward polyploid evolution. The phylogenetic relationship between *Z. armatum* ‘Tengjiao’ and *Z. armatum* var. *novemfolius* was the closest, while that between *Z. armatum* ‘YT-03’ and *Z. armatum* ‘YT-06’ was closer than with *Z. armatum* ‘Youkangtengjiao’ according to oligo-FISH. The results provided a karyotype profile and a physical map that contributes to the distinction of varieties and cultivars of *Z. armatum* and provides strategies for distinguishing other cultivated species.

## 1. Introduction

*Zanthoxylum* is a genus of economically important aromatic plants in the family Rutaceae, commonly referred to as Sichuan pepper [1,2], which has over 200 species [3]. Currently, there are 45 species and 13 varieties of wild *Zanthoxylum* in China [4]. *Zanthoxylum* has been bred for more than 2000 years in China [5]. In addition to being used in cooking, *Zanthoxylum* is used as an insect repellent and as a medicinal treatment for fever, Alzheimer's disease, indigestion, and stomach diseases [6,7]. Compared with *Zanthoxylum bungeanum* Maxim, *Zanthoxylum armatum* DC has a unique aroma that gradually came to be favored by consumers who preferred it to the numbing taste of *Z. bungeanum* [8]. In the Sichuan province, the *Z. armatum* planting area has exceeded 2000 square kilometers [9]. The Chinese agricultural industry of *Z. bungeanum* and *Z. armatum* is worth more than USD 4 billion [10]. In some countries and regions, cultivation of *Zanthoxylum* as a medicinal plant has been prioritized to help improve local economies [11,12]. Sichuan is rich in varieties of *Z. armatum*, featuring many high-quality cultivars that are products of long-term domestication [13]. The flower organs and fruits of various varieties and cultivars of *Z. armatum* are similar, which makes them difficult to distinguish using traditional morphological classification methods.

Compared to other traits, plants’ chromosomal features are relatively stable [14]. Chromosome numbers are widely documented because they are relatively easy to determine and catalog [15]. Chromosome numbers can be used to study phylogenetic characteristics and infer genomic events [16]. As *Zanthoxylum* has many chromosomes, it is difficult to determine its chromosome number; the chromosome numbers of many *Zanthoxylum* species are still debated [3]. *Z. acanthopodium* Candelle has 2*n* = 64 chromosomes, *Z. dimorphophyllum* Hemsley has 2*n* = 36 and 68 chromosomes [17], *Z. scandens* Blume has 2*n* = 68 chromosomes [17], *Z. oxyphyllum* Edgeworth has 2*n* = 72 chromosomes [17], *Z. tomentellum* J.D. Hance has 2*n* = 72 chromosomes [17], *Z. simulans* Hance has 2*n* = 132 chromosomes [17], *Z. nitidum* (Roxburgh) Candolle has 2*n* = 68 chromosomes [18], and *Z. bungeanum* has 2*n* = 136 chromosomes [10,19,20,21]. Previously recorded chromosome numbers for *Z. armatum* include 2*n* = 66 [17], ~128 [3], 132 [20], and 136 [16]; the chromosome numbers are indeterminate and numerous. Chromosome research requires a physical chromosome map [22]; however, a chromosome map’s development is often hampered by a lack of markers that allow the identification of individual chromosomes. Many techniques have been applied to overcome this barrier [23]. The fluorescence in situ hybridization of oligonucleotides (oligo-FISH) using chromosome-specific probes to construct a physical chromosome map [24] is widely used to determine genetic relationships among plant species [25,26] as well as in phylogenetic reconstruction [27] and molecular cytology [28]. Different combinations of oligonucleotide probes have been used to study cultivars. For example, a combination of 5S rDNA and (AG_3_T_3_)_3_ probes was used to distinguish *Hippophae rhamnoides* L. and Sichuan walnut cultivars [29,30]. By utilizing oligo-FISH technology in conjunction with various oligonucleotide-specific probes, it is possible to distinguish between different varieties and cultivars of species that possess similar morphologies.

The chromosome numbers of varieties and cultivars of *Z. armatum* are not clear, and ploidy is controversial. This was the first study to use three oligonucleotide probes to distinguish varieties and cultivars of *Z. armatum*. We used three oligonucleotide probes to study five varieties and cultivars of *Z. armatum* and analyzed their relationship according to the number of signals. We found that the number of chromosomes within the *Z. armatum* species was inconsistent, and the specific oligonucleotide probes of the materials within the *Z. armatum* species showed signal differences, intuitively showing the different genetic relationships among varieties and cultivars. The results of this study can be helpful for the identification of varieties and cultivars of *Z. armatum* in the future.

## 2. Materials and Methods

### 2.1. Plant Materials

The seedlings were collected from a cultivar garden in Zigong City, Sichuan Province. The materials are shown in Table 1. *Z. armatum* ‘Tengjiao’ and *Z. armatum* var. *novemfolius* are famous cultivars of *Z. armatum* with a huge planting area in southwest China [9]. *Z. armatum* ‘Youkangtengjiao’, *Z. armatum* ‘YT-03’, and *Z. armatum* ‘YT-06’ are excellent varieties of *Z. armatum* with special disease resistance and a high yield.

These materials in the cultivar garden were managed at the same level and sampled at the same time. Five seedlings were transplanted to the Chengdu Academy of Agriculture and Forestry Sciences and photographed with a stereomicroscope after normal growth. When the root tip grew to 1.5–2 cm, the root-tip meristem was removed, placed in nitrous oxide for 2.5 h, soaked in acetic acid for 5 min, and then stored in 75% ethanol at −20 °C.

### 2.2. FISH

The root-tip meristems were dispersed in cellulose and pectinase (2:1) and then maintained in this mixture at 37 °C for 45 min, as described by Luo et al. [29]. The enzyme mixture was subsequently washed using ddH_2_O twice. The ddH_2_O was then removed from the mixture, and the mixture was subsequently washed in ethyl alcohol twice. After it air-dried completely, 20 µL of glacial acetic acid was added; then, the enzyme mixture was dropped onto a clean slide and examined using an Olympus CX23 microscope (Olympus Corporation, Tokyo, Japan). This study used (AG_3_T_3_)_3_ [31], 5S rDNA [14], and (GAA)_6_ [3] as probes; they were produced by Sangon Biotechnology Co., Ltd. (Shanghai, China). The 5’ ends of the probe were labeled using 6-carboxyfluorescein (FAM) or 6-carboxytetramethylrhodamine (TAMRA). The synthetic probes were dissolved in 1× Tris-ethylenediaminetetraacetic acid (TE) at a concentration of 10 µM and stored at −20 °C.

The slides were subjected to chromosome fixation, dehydration, and denaturation before being dehydrated again and then hybridized using the denatured probe mixture at 37 °C for 1.5 h [3]. After hybridization, 10 µL of 4,6-diamidino-2-phenylindole (DAPI) was dropped onto slides for observation using an Olympus BX63 fluorescence microscope combined with a Photometric SenSys Olympus DP70 CCD camera (Olympus Corporation, Tokyo, Japan). Signal patterns were analyzed using the three best spreads. The length of each chromosome was calculated using Photoshop version 2021 (Adobe Systems Inc., San Jose, CA, USA); each spread was measured three times to obtain an average value and consistent chromosome data. Karyotype data (karyotype formula, arm ratio, and karyotype type) were determined using NucType 2013; the analysis was based on the length of each chromosome. The physical map was generated on the basis of the signals of 5S rDNA and (GAA)_6_ using Photoshop version 2021.

### 2.3. Genetic Relationships

The study utilized 5S rDNA- and (GAA)_6_-specific oligonucleotide probes to analyze the genetic relationships among five *Z. armatum* materials. The 5S rDNA signals were used as the primary reference basis for phylogenetic analysis; these were previously used in various species. To determine the genetic relationship, the number of 5S rDNA signals was screened; close numbers indicated a close relationship. If the number of 5S rDNA signals was consistent, (GAA)_6_ was used as an auxiliary reference. The physical map of *Z. armatum* was created using Photoshop version 2021.

## 3. Results

### 3.1. Flower Bud and Fruit Morphology of Z. armatum

**Figure 1:**
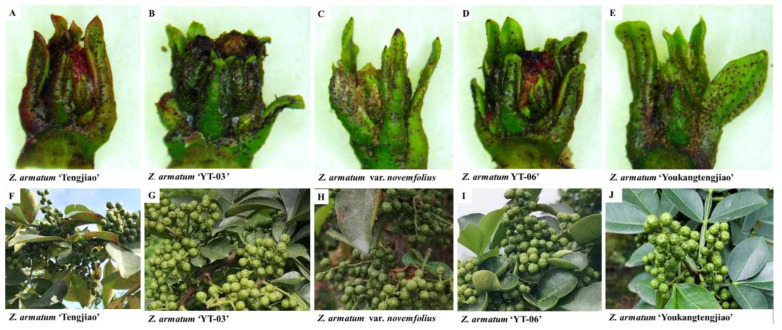
Five varieties and cultivars of *Z. armatum*’ flower bud and fruit morphologies. Flower bud samples were collected during germination, and images were taken using a stereomicroscope. Fruit morphology images were taken using a camera. (**A**) Flower bud of *Z. armatum* ‘Tengjiao’, (**B**) flower bud of *Z. armatum* ‘YT-03’, (**C**) flower bud of *Z. armatum* var. *novemfolius*, (**D**) flower bud of *Z. armatum* ‘YT-06’, (**E**) flower bud of *Z. armatum* ‘Youkangtengjiao’, (**F**) fruit of *Z. armatum* ‘Tengjiao’, (**G**) fruit of *Z. armatum* ‘YT-03’, (**H**) fruit of *Z. armatum* var. *novemfolius*, (**I**) fruit of *Z. armatum* ‘YT-06’, and (**J**) fruit of *Z. armatum* ‘Youkangtengjiao’.

The phenotypic similarity of the flower buds and fruits of the five varieties and cultivars of *Z. armatum* was evaluated. Compared with the flower bud stage, the bud at the bottom of *Z. armatum* ‘Tengjiao’ had little morphological change, and the bud at the top of *Z. armatum* ‘Tengjiao’ became round and long in shape. In the early quilt differentiation stage of flower bud differentiation of *Z. armatum*, the flower bud development degree of *Z. armatum* ‘Tengjiao’ was slightly lower than that of other varieties and cultivars of *Z. armatum*. The upper end of the flower bud of *Z. armatum* var. *novemfolius* became round and large, and the young leaves covering the flower bud began to stretch while the bud became spherical, reaching the late stage of ovary differentiation of *Z. armatum* with the fastest development degree. The other varieties and cultivars of *Z. armatum* were in the early stage of ovary differentiation, and the flower buds became slightly round to spherical in shape. The fruit morphologies of the five varieties and cultivars of *Z. armatum* were similar and difficult to distinguish. The flower bud and fruit morphologies of the five materials of *Z. armatum* are shown in Figure 1.

### 3.2. Karyotype of Z. armatum

**Figure 2:**
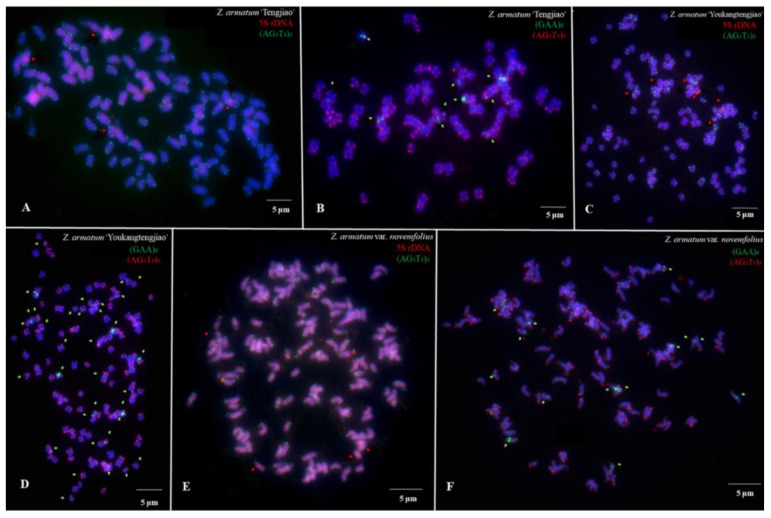
Mitotic metaphase chromosome fluorescence in situ hybridization (FISH) visualization of *Z. armatum*. (**A**) *Z. armatum* ‘Tengjiao’ using (AG_3_T_3_)_3_ (green) and 5S rDNA (red) probes, (**B**) *Z. armatum* ‘Tengjiao’ using (AG_3_T_3_)_3_ (red) and (GAA)_6_ (green) probes, (**C**) *Z. armatum* ‘Youkangtengjiao’ using (AG_3_T_3_)_3_ (green) and 5S rDNA (red) probes, (**D**) *Z. armatum* ‘Youkangtengjiao’ using (AG_3_T_3_)_3_ (red) and (GAA)_6_ (green) probes, (**E**) *Z. armatum* var. *novemfolius* using (AG_3_T_3_)_3_ (green) and 5S rDNA (red) probes, and (**F**) *Z. armatum* var. *novemfolius* using (AG_3_T_3_)_3_ (red) and (GAA)_6_ (green) probes. The red signal was 6-carboxytetramethylrhodamine (TAMRA), while the green signal was 6-carboxyfluorescein (FAM). Chromosomes were counterstained using 4,6-diamidino-2-phenylindole (DAPI) (blue). Scale bar = 5 µm.

**Figure 3:**
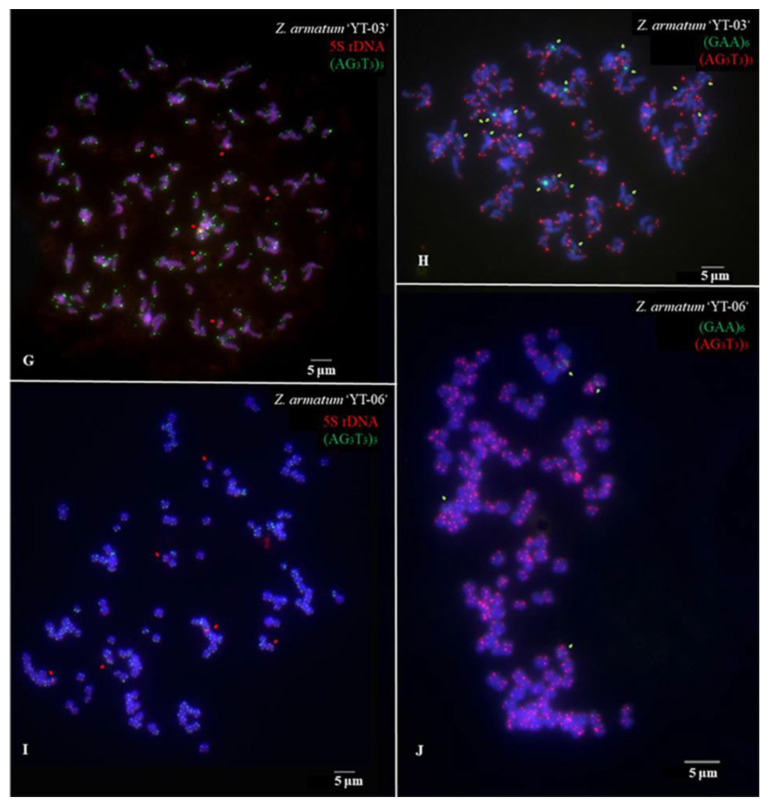
Mitotic metaphase chromosomes fluorescence in situ hybridization (FISH) visualization of *Z. armatum*. (**G**) *Z. armatum* ‘YT-03’ using (AG_3_T_3_)_3_ (green) and 5S rDNA (red) probes, (**H**) *Z. armatum* ‘YT-03’ using (AG_3_T_3_)_3_ (red) and (GAA)_6_ (green) probes, (**I**) *Z. armatum* ‘YT-06’ using (AG_3_T_3_)_3_ (green) and 5S rDNA (red) probes, and (**J**) *Z. armatum* ‘YT-06’ using (AG_3_T_3_)_3_ (red) and (GAA)_6_ (green) probes. The red signal was 6-carboxytetramethylrhodamine (TAMRA), while the green signal was 6-carboxyfluorescein (FAM). Chromosomes were counterstained using 4,6-diamidino-2-phenylindole (DAPI) (blue). Scale bar = 5 µm.

**Figure 4:**
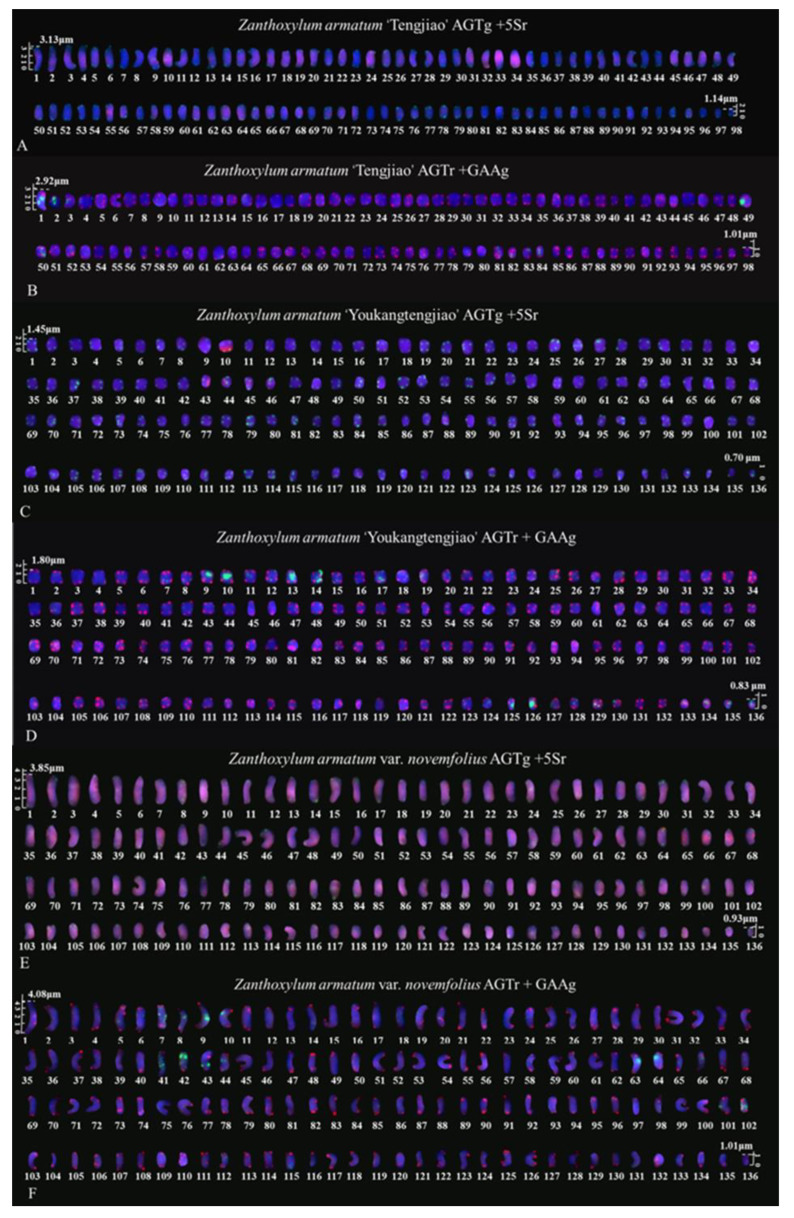
Mitotic chromosomes of *Z. armatum* rearranged from Figure 2. (**A**) *Z. armatum* ‘Tengjiao’ using (AG_3_T_3_)_3_ (green) and 5S rDNA (red) probes, (**B**) *Z. armatum* ‘Tengjiao’ using (AG_3_T_3_)_3_ (red) and (GAA)_6_ (green) probes, (**C**) *Z. armatum* ‘Youkangtengjiao’ using (AG_3_T_3_)_3_ (green) and 5S rDNA (red) probes, (**D**) *Z. armatum* ‘Youkangtengjiao’ using (AG_3_T_3_)_3_ (red) and (GAA)_6_ (green) probes, (**E**) *Z. armatum* var. *novemfolius* using (AG_3_T_3_)_3_ (green) and 5S rDNA (red) probes, and (**F**) *Z. armatum* var. *novemfolius* using (AG_3_T_3_)_3_ (red) and (GAA)_6_ (green) probes. Chromosomes were aligned according to length from longest to shortest. The scale bars range from 0.5 to 5 μm.

**Figure 5:**
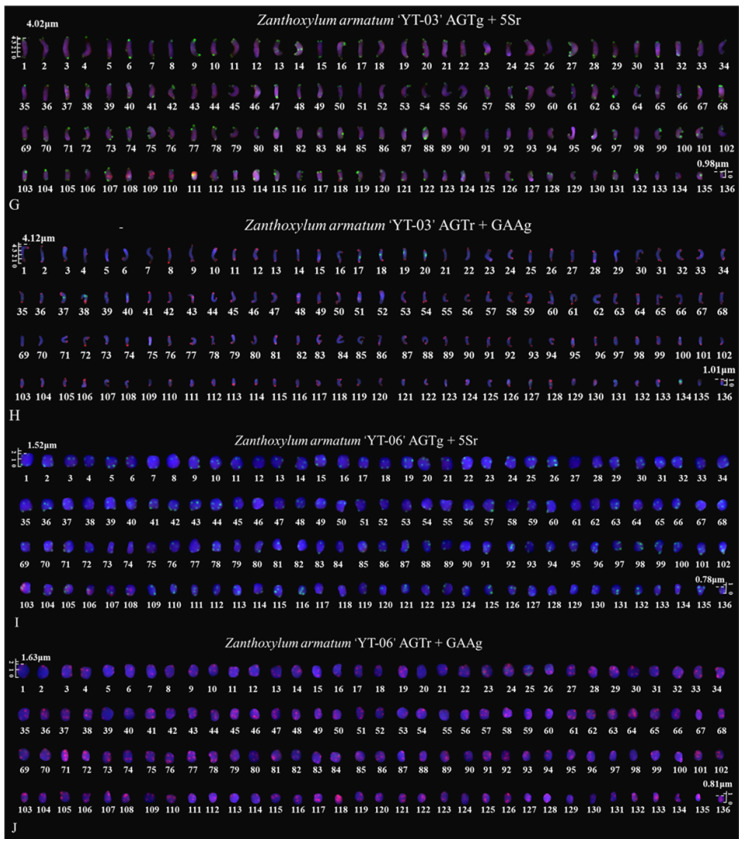
Mitotic chromosomes of *Z. armatum* rearranged from Figure 3. (**G**) *Z. armatum* ‘YT-03’ using (AG_3_T_3_)_3_ (green) and 5S rDNA (red) probes, (**H**) *Z. armatum* ‘YT-03’ using (AG_3_T_3_)_3_ (red) and (GAA)_6_ (green) probes, (**I**) *Z. armatum* ‘YT-06’ using (AG_3_T_3_)_3_ (green) and 5S rDNA (red) probes, and (**J**) *Z. armatum* ‘YT-06’ using (AG_3_T_3_)_3_ (red) and (GAA)_6_ (green) probes. Chromosomes were aligned according to length from longest to shortest. The scale bars range from 0.5 to 5 μm.

The metaphase chromosomes of *Z. armatum* were analyzed using FISH, as shown in Figure 2 and Figure 3. The chromosome number of *Z. armatum* ‘Tengjiao’ was 98, while the chromosome number of *Z. armatum* ‘Youkangtengjiao’, *Z. armatum* var. *novemfolius*, *Z. armatum* ‘YT-03’, and *Z. armatum* ‘YT-06’ was 136. To better characterize these chromosomes, individual chromosomes were aligned according to length from the longest to the shortest, as illustrated in Figure 4 and Figure 5. 

The karyotype formula of *Z. armatum* ‘Tengjiao’ was 2*n* = 2*x* = 98*m* with karyotype type 1C and an arm ratio of 4.3237. The karyotype formula of *Z. armatum* ‘Youkangtengjiao’ was 2*n* = 2*x* = 128*m* + 8*sm* with karyotype type 2B and an arm ratio of 3.5336. The karyotype formula of *Z. armatum* var. *novemfolius* was 2*n* = 2*x* = 134*m* + 2*sm* with karyotype type 1C and an arm ratio of 5.5224. The karyotype formula of *Z. armatum* ‘YT-03’ was 2*n* = 2*x* = 2*M* + 128*m* + 4*sm* + 2*st* with karyotype type 2C and an arm ratio of 4.1829. The karyotype formula of *Z. armatum* ‘YT-06’ was 2*n* = 2*x* = 126*m* + 10*sm* with karyotype type 2B and an arm ratio of 3.3011. The chromosomal symmetry of *Z. armatum* 'Tengjiao' was high, whereas the chromosomal symmetry of *Z. armatum* 'YT-03' was low, with the karyotypes of the five materials showing a trend toward polyploid evolution. The karyotypes of the five varieties and cultivars of *Z. armatum* are shown in Table 2.

### 3.3. Oligonucleotide Signal Number of Different Z. armatum Materials

(AG_3_T_3_)_3_ signals were located at all chromosome ends of both sample materials at the centromeres and proximally. (AG_3_T_3_)_3_ signals were difficult to distinguish but helped in counting the chromosomes in the five varieties and cultivars of *Z. armatum*; 5S rDNA and (GAA)_6_ signals had different locations and numbers in the five varieties and cultivars of *Z. armatum.*

**Figure 6:**
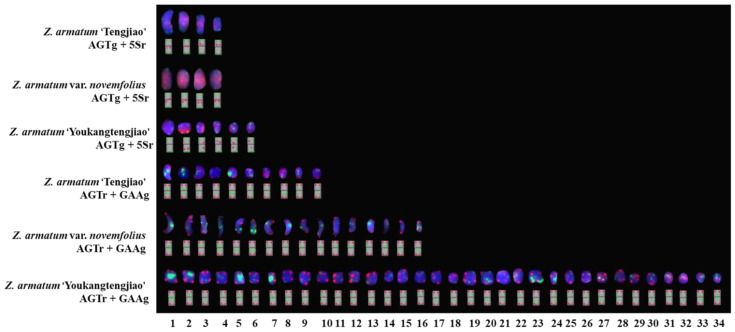
Physical map of *Z. armatum*. Signal pattern ideographs were constructed on the basis of the signal patterns of the chromosomes mentioned above as well as the chromosomes in Figure 2 and Figure 3. The number of chromosomes is indicated across the bottom. Signal combinations noted to the left were consistent with the findings in Figure 2 and Figure 3.

To clarify the signal distribution of *Z. armatum*, we created a physical chromosomal map, as shown in Figure 6. *Z. armatum* ‘Youkangtengjiao’ had the most 5S rDNA and (GAA)_6_ signals. *Z. armatum* ‘YT-06’ had the fewest (GAA)_6_ and 5S rDNA signals. *Z. armatum* ‘Tengjiao’ and *Z. armatum* var. *novemfolius* had two pairs of 5S rDNA signals. *Z. armatum* ‘Youkangtengjiao’, *Z. armatum* ‘YT-03’, and *Z. armatum* ‘YT-06’ had three pairs of 5S rDNA signals. *Z. armatum* ‘YT-06’ had two pairs of (GAA)_6_ signals. *Z. armatum* ‘Tengjiao’ had five pairs of (GAA)_6_ signals. *Z. armatum* var. *novemfolius* had eight pairs of (GAA)_6_ signals. *Z. armatum* ‘YT-03’ had nine pairs of (GAA)_6_ signals. *Z. armatum* ‘Youkangtengjiao’ had 17 pairs of (GAA)_6_ signals.

The phylogenetic relationship between *Z. armatum* ‘Tengjiao’ and *Z. armatum* var. *novemfolius* was the closest, while that between *Z. armatum* ‘YT-03’ and *Z. armatum* ‘YT-06’ was closer than with *Z. armatum* ‘Youkangtengjiao’ according to 5S rDNA and (GAA)_6_ signals. The phylogenetic relationships of the five *Z. armatum* materials were also confirmed by the karyotype analysis (Table 2). The strong symmetry of *Z. armatum* ‘Tengjiao’ and *Z. armatum* var. *novemfolius* indicated that their divergence was minimal.

## 4. Discussions

### 4.1. Inconsistent Chromosome Number of Five Z. armatum Materials

Chromosomal data are fundamental to cytological studies [32] and widely documented in most families and genera [15]. Chromosomal data are useful for solving traditional plant classifications [31]; moreover, because they are relatively stable in plants, they can also be used to study chromosome aberration, cell function, and inference of genomic events [14,16].

Previously, *Z. armatum* was reported to have 2*n* = 66 [17], ~128 [3], 132 [20], and 136 chromosomes [16], which differed from our results. Our chromosome number results for the five varieties and cultivars of *Z. armatum* were inconsistent with those of Zhang and Hartley [17]; this may be because Zhang and Hartley [17] adopted the traditional production method. Their process for obtaining chromosomes was rough, and some chromosomes may have been washed out. Additionally, chromosomes overlapped during production, which may have caused the chromosome numbers to differ. Our results for *Z. armatum* ‘YT-03’, *Z. armatum* ‘YT-06’, *Z. armatum* ‘Youkangtengjiao’, and *Z. armatum* var. *novemfolius* were inconsistent with Luo et al.’s results for two possible reasons. First, different research materials were used. Luo et al. [3] used *Z. armatum* ‘Hanyuanputaoqing’ instead of our research cultivars of *Z. armatum*. Thus, Luo et al.’s [3] research results might indirectly prove that different cultivars of *Z. armatum* have different chromosome numbers. Their *Z. armatum* cultivars were ambiguous, and the materials themselves were problematic [33]. Second, Luo et al. [3] did not use telomere (AG_3_T_3_)_3_ probes to ensure chromosome integrity and counting accuracy, which may have resulted in counting errors. Hu et al. [20] used telomere probes, but some chromosomes were superimposed in the division phase, which could have easily led to miscounting. Secondly, their research did not specify the name of the *Z. armatum* cultivars, which may have differed from the materials we used.

*Z. armatum* ‘Youkangtengjiao’ was bred from *Z. armatum* ‘Tengjiao’; they belong to the same species and should have the same number of chromosomes. However, we found an interesting phenomenon; i.e., their chromosome numbers were inconsistent. We hypothesize that the reason for this might be related to the apomictic reproduction of *Z. armatum*. Apomictic reproduction does not require parental gametophytes to produce zygotes; offspring are produced directly from the mother [34]. This method of reproduction could fix the mother’s economic traits, which would be beneficial for breeding [35]. It is possible that apomictic reproduction led to similar phenotypic characteristics in *Z. armatum*, although there were many varieties and cultivars. Apomixis may have formed nested chromosome fusions (NCF), leading to changes in chromosome numbers. The vast majority of cases were polyploid, which resulted in abnormal chromosome numbers [36]. This phenomenon was previously found in wheat [37], *Coccinia grandis* (L.) Voigt [38], and grapevine varieties [39]. The *Z. armatum* genus genome is large; extensive transposable element (TEs) and events of whole-genome duplication (WGD) led to changes in the plant genome [40,41,42]. Active TEs might have triggered fission and fusion events of *Z. armatum* chromosomes [43], which might have led to changes in the chromosome number [44]. Understanding chromosome numbers is beneficial for future *Z. armatum* gene assembly and would allow a physical map of oligonucleotides to be drawn. In the future, we will identify the chromosomes of other varieties and cultivars of *Z. armatum.*

### 4.2. Divergence Stages of Five Z. armatum Materials

Karyotype analysis can not only study the phylogenetic relationship but also provide evidence for the cell research of this species [35]. In this study, the karyotype data of five varieties and cultivars of *Z. armatum* were inconsistent, which may be related to the region and growing environment [45]. *Z. armatum* ‘Tengjiao’ grows in the rainy zone of West China, while *Z. armatum* var. *novemfolius* grows in a subtropical monsoon humid climate [46]. Plants living in different environments for a long time will not only have divergent adaptation but will also undergo genetic changes [47], thus influencing chromosome data. The inconsistent karyotype analysis data may also be related to the difficulty of karyotype analysis due to the small chromosome size of *Z. armatum* [3]. In the karyotype analysis of five varieties and cultivars of *Z. armatum*, some centromeric points were not obvious, and there was difficultly in the karyotype analysis. We conducted the karyotype analysis on the basis of the clearest centromeric points. The inconsistent karyotype data of the five varieties and cultivars of *Z. armatum* may also be related to the apomictic reproduction of *Z. armatum*, which resulted in nested chromosome fusion, leading to changes in the number of chromosome karyotypes. At the same time, *Z. armatum* has a large genome, and the occurrence of TEs and WGD can also lead to gene changes, which may alter the chromosome karyotype data. The genome of *Z. armatum* is still undetermined despite some studies [8,10]; thus, further studies are needed to determine how genetic changes affect the chromosome.

Stebbins et al. [48] believed that a higher chromosome symmetry indicates earlier divergence. The evolutionary trend of karyotype ranges from symmetrical to asymmetrical. The chromosomes of the five varieties and cultivars of *Z. armatum* were mainly *m* and *sm* chromosomes with a small number of *M* and *st* chromosomes. Overall, the five varieties and cultivars of *Z. armatum* had high symmetry, possibly indicating early divergence. The *m* chromosome of *Z. armatum* ‘Tengjiao’ was the least evolved. *Z. armatum* ‘YT-03’ had lower symmetry and was more evolved. Some plant karyotypes can evolve from diploid to polyploid [49]. The evaluation of the five materials showed that the karyotype of varieties and cultivars of *Z. armatum* also potentially evolved from diploid to polyploid. *Z. armatum* ‘Tengjiao’ can maintain a relatively primitive state, which may be related to the stability of the current environment [45]. At present, the climate is relatively stable and there is no extreme climate; hence, rattan pepper has adapted to the local climate environment with a low degree of evolution.

The chromosome of *Z. armatum* can be considered small [3], complicating karyotype analysis. Although our study could not accurately describe the karyotype data of *Z. armatum*, it could reflect the genetic differences at the chromosome level among the five varieties and cultivars of *Z. armatum*. The evolutionary relationship of the different varieties and cultivars of *Z. armatum* requires further study.

### 4.3. Oligonucleotide Signal Differences of Five Z. armatum Materials

Using single-copy and repetitive DNA sequences of plants and chromosome-specific probes [50], FISH is a valuable molecular cytogenetic mapping tool, especially for species with small chromosome numbers and similar morphologies [51]. The numbers and distribution of chromosomes according to FISH can reveal genetic composition patterns and karyotype variations [52]. Oligo-FISH can be used to analyze karyotypes, chromosome rearrangements, and meiotic pairing [53], and it has been widely used in determining genetic relationships among plant species [25,26], as well as in phylogenetic reconstruction [27] and molecular cytology [28], with rapid and efficient results [32].

It is known that 5S rDNA may be present at the middle, near middle, and end of each chromosome [14,54]. There is a strong relationship between signal number and ploidy [55]. To date, FISH has identified the number and location of rDNA loci in more than 1600 plant species [56]. The quantity and location of 5S and 45S rDNA are often characteristic of a particular species or genus [57,58,59]. As a powerful cytogenetic marker, 5S rDNA [60] is helpful in the study of reproductive segregation and speciation [61], chromosome characterization [62], and genome differentiation [27]. Using FISH technology to locate repetitive DNA sequences helps correct the karyotypes of small chromosomes and insignificant morphological characteristics, which can reflect the degree of differentiation between species and genera at the chromosome level [54,63]. Luo et al. [3] obtained two pairs of 5S rDNA signal sites, which was consistent with *Z. armatum’s* 5S rDNA signal sites identified in this study but inconsistent with the three pairs of 5S rDNA sites of *Z. armatum* ‘Youkangtengjiao’, *Z. armatum* ‘YT-03’, and *Z. armatum* ‘YT-06’. We speculated that this may be because *Z. armatum* ‘Youkangtengjiao’, *Z. armatum* ‘YT-03’, and *Z. armatum* ‘YT-06’ had new chromosomes containing more genetic information. The signal locations of *Z. armatum* ‘Tengjiao’ and *Z. armatum* var. *novemfolius’* were the same as those found by Luo et al. [3]; this may indicate that the two cultivars share similar genetic information.

The (AG_3_T_3_)_3_ signals may be present in the proximal, distal, and dissociative parts of chromosomes [29]. The (AG_3_T_3_)_3_ probe was used to determine the integrity and number of chromosomes because it is often present at both ends of chromosomes [29]. The (AG_3_T_3_)_3_ probe was used herein for the first time in *Z. armatum*. The probe signal sites appeared at both ends of the chromosome, which is convenient for counting chromosomes. GAA signals may appear in the proximal telomeric, sub-telomeric, and interstitial regions [64,65,66,67]; this is beneficial for cytogenetics and genome organization research. Luo et al. [3] found five pairs of (GAA)_6_ signals in *Z. armatum*, which was consistent with our results for *Z. armatum* ‘Tengjiao’ but inconsistent with the 17 pairs of (GAA)_6_ signals in *Z. armatum* ‘Youkangtengjiao’, the two pairs of (GAA)_6_ signals in *Z. armatum* ‘YT-03’*,* the nine pairs of (GAA)_6_ signals in *Z. armatum* ‘YT-06’, and the eight pairs of (GAA)_6_ signals in *Z. armatum* var. *novemfolius*.

Using signal numbers and locations, we were able to find genetic relationships. For example, *Z. armatum* ‘Tengjiao’ and *Z. armatum* var. *novemfolius* were the closest, whereas *Z. armatum* ‘YT-03’ and *Z. armatum* ‘YT-06’ were closer than *Z. armatum* ‘Youkangtengjiao’.

### 4.4. Distinction of Varieties and Cultivars of Z. armatum based on Chromosome Number and Oligonucleotide Signal

We observed that the chromosome number of *Z. armatum* ‘YT-03’, *Z. armatum* ‘YT-06’, *Z. armatum* ‘Youkangtengjiao’, and *Z. armatum* var. *novemfolius* was 136, whereas that of *Z. armatum* ‘Tengjiao’ was 98. Accordingly, we directly determined that *Z. armatum* ‘Tengjiao’ differed from other varieties and cultivars of *Z. armatum*. The chromosome number of *Z. armatum* ‘YT-03’, *Z. armatum* ‘YT-06’, *Z. armatum* ‘Youkangtengjiao’, and *Z. armatum* var. *novemfolius* was the same, but their 5S rDNA and (GAA)_6_ probe signals were different. *Z. armatum* ‘Youkangtengjiao’ had three pairs of 5S rDNA signals and 17 pairs of (GAA)_6_ signals. *Z. armatum* var. *novemfolius* had two pairs of 5S rDNA signals and eight pairs of (GAA)_6_ signals. *Z. armatum* ‘YT-03’ had three pairs of 5S rDNA signals and nine pairs of (GAA)_6_ signals. *Z. armatum* ‘YT-06’ had three pairs of 5S rDNA signals and two pairs of (GAA)_6_ signals. We distinguished varieties and cultivars of *Z. armatum* using probe signals. In addition, different chromosome lengths represent an auxiliary basis for distinguishing varieties and cultivars of *Z. armatum*.

It is difficult to distinguish intra- and interspecific differences using only FISH [30]. To completely distinguish intra- and interspecific differences, it is necessary to design sufficient probes and draw oligonucleotide maps. However, because of the large number of chromosomes and unclear genome sequences, this work will be extremely challenging. Some chromosomal changes (deletions, duplications, inversions, or translocations) [30] and B chromosome generation [15,50] can result in changes in the number of chromosomes and more complex genetic information. However, woody plant genomes are highly heterozygous and have a complex genetic background [68]. In the Rutaceae family, complete genome sequences have been studied only in *Citrus* [1,2,69]. Although there have been some studies of the *Zanthoxylum* genome [8,10,36], they had pre-existing limitations. Therefore, more in-depth research is needed.

## 5. Conclusions

There were differences in the karyotypes of the five varieties and cultivars of *Z. armatum*, showing a trend toward polyploid evolution. Our research method can be used to distinguish different varieties and cultivars of *Z. armatum* as well as to provide a reference for the future cultivation of different species.

## Figures and Tables

**Table 1:** Five varieties and cultivars of *Z. armatum*.

No.	Latin Name	Type	Location	Geographical Coordinates
1	*Z. armatum* ‘Tengjiao’	Seedling	Zigong City, Sichuan Province, China	104°52′10″ E, 29°16′28″ N
2	*Z. armatum* ‘Youkangtengjiao’	Seedling	Zigong City, Sichuan Province, China	104°52′10″ E, 29°16′28″ N
3	*Z. armatum* var. *novemfolius*	Seedling	Zigong City, Sichuan Province, China	104°52′10″ E, 29°16′28″ N
4	*Z. armatum* ‘YT-03’	Seedling	Zigong City, Sichuan Province, China	104°52′10″ E, 29°16′28″ N
5	*Z. armatum* ‘YT-06’	Seedling	Zigong City, Sichuan Province, China	104°52′10″ E, 29°16′28″ N

**Table 2:** Karyotype analysis of five varieties and cultivars of *Z. armatum*.

No.	Latin Name	Karyotype	Karyotype Type	Arm Ratio
1	*Z. armatum* var. *novemfolius*	2*n* = 2*x* = 134*m* + 2*sm*	1C	5.5224
2	*Z. armatum* ‘Tengjiao’	2*n* = 2*x* = 98*m*	1C	4.3237
3	*Z. armatum* ‘YT-03’	2*n* = 2*x* = 2*M* + 128*m* + 4*sm* + 2*st*	2C	4.1829
4	*Z. armatum* ‘Youkangtengjiao’	2*n* = 2*x* = 128*m* + 8*sm*	2B	3.5336
5	*Z. armatum* ‘YT-06’	2*n* = 2*x* = 126*m* + 10*sm*	2B	3.3011

## Data Availability

Not applicable.
